# Dairy Intake Would Reduce Nutrient Gaps in Chinese Young Children Aged 3–8 Years: A Modelling Study

**DOI:** 10.3390/nu12020554

**Published:** 2020-02-20

**Authors:** Xiaofang Jia, Dantong Wang, Alison L. Eldridge, Bing Zhang, Xiaofan Zhang, Huijun Wang

**Affiliations:** 1 National Institute for Nutrition and Health, Chinese Center for Disease Control and Prevention, Beijing 100050, China; jiaxf@ninh.chinacdc.cn (X.J.); zhangbing@chinacdc.cn (B.Z.); zhangxf@ninh.chinacdc.cn (X.Z.); 2 Nestlé Research Center, CH-1000 Lausanne, Switzerland; dantong.wang@rdls.nestle.com (D.W.); alison.eldridge@rdls.nestle.com (A.L.E.)

**Keywords:** dairy consumption, formulated milk powder for children ≥3 years, milk, nutrient inadequacy, dietary modelling

## Abstract

Dairy foods are under-consumed among Chinese children. We modeled the impact of increased dairy consumption on nutrient inadequacy and assessed whether the consumption of formulated milk powder for children ≥3 years (FMP3+) is useful for reducing nutrient gaps. Data from 3–8-year-old children, with completed socio-demographic and dietary measurements from the China Health and Nutrition Survey 2015, were used (*n* = 1122). Dietary intakes were modeled in two scenarios: Scenario 1 added FMP3+ or cow’s milk to reported diet to reach recommended dairy intakes; Scenario 2 replaced the currently consumed milk with an equal volume of FMP3+. Reported nutrient intakes were compared with each model. Only 32.5% of children consumed dairy products; the average intake amount in total was 48.6 g/day. Most children (97.6%) did not meet dairy intake recommendation. Inadequate nutrient intakes were observed for calcium, potassium, thiamin, riboflavin, vitamin C and selenium. In Scenario 1, both FMP3+ and cow’s milk improved the intake of all analyzed nutrients. In Scenario 2, FMP3+ substitution increased the intake of most nutrients, and reduced the proportion of children with an inadequate intake of vitamin C, thiamin, vitamin A, iron, zinc and potassium. Thus, increasing dairy consumption would reduce nutrient gaps, and FMP3+ is a good food source to help children meet nutrient requirements.

## 1. Introduction

The diet quality of children has both short-term and long-term effects on health [1]. Children require sufficient nutrients to meet the need for rapid growth and development [2]. Many studies around the world focus on evaluating nutrient intakes and their adherence to recommended reference values among young children [3,4,5,6]. Bailey et al. [3] reported that 79% and 30% of 3–4-year-old US children consumed less than the estimated average requirement (EAR) for vitamins D and E, respectively, although their intakes of most other nutrients were largely adequate. These children also consumed excessive amounts of sodium (75%) and zinc (69%). Nutrient intakes and compliance with dietary reference intakes are varied among European countries. For example, in Spanish children aged 4–8 years, 42.9% girls were below the lower limit of the acceptable macronutrient distribution ranges (AMDR) for carbohydrate, and intakes of vitamin D, vitamin E, folate and calcium were below recommendations for both genders [7,8]. The situation of nutrient intake in young children in Asia is quite different from western countries. In the Philippines, the intake of total fat as a percentage of energy, and most micronutrients, were highly inadequate due to poor diet quality for children aged 3–6 years, with inadequacies in iron (90%), calcium (84%), folate (72%), vitamin C (60%), zinc (47%) [4]. In China, micronutrient inadequacy is a significant nutritional issue in children and adolescents aged 3–17 years, with low intakes of calcium, selenium, thiamin, riboflavin and vitamin C compared to the recommendations [9,10]. Variations in dietary habits could result in differences in nutrient intakes. Poor diet quality is directly associated with nutrient inadequacy [9].

The dietary patterns of children 2–5 years old become close to that of adults [1,11]. In order to fulfill the nutritional requirements for growth and development, young children should have a nutritionally adequate and safe diet [12]. The Dietary Guidelines for Chinese, published in 2016, provides intake recommendations for each food group for healthy individuals aged 2 years and above [11]. These guidelines include a recommendation to consume dairy products daily, equivalent to 300 g/day fluid milk, which is an important food source for dietary calcium, vitamin D, potassium, protein, zinc and other nutrients. However, dairy product consumption is low in the Chinese population [13]. In 2006, only 14.1% of Chinese 7–11 year old children reported consuming dairy products over three days of dietary recalls, and the average amount consumed was 25.1 g/day [14]. Large disparities were observed in the percent of children consuming milk and dairy products by dwelling location (70.4% in highly urban areas vs. 14.8% in rural areas) among Chinese children aged 4–17 years in 2011 [15].

Formulated milks are milk-based products fortified with nutrients that can be lacking in the general diets of children [16,17]. Two clinical trials have shown improvements in vitamin D and iron status in young children following interventions with formulated milk compared with cow’s milk, but other nutrients were not evaluated [18,19]. Several modelling studies of dairy food consumption in US children have been conducted to assess the impact of increasing dairy consumption on nutrient intakes [20,21,22], but these studies did not evaluate formulated milk. In addition, a modelling study in the UK showed that the replacement of cow’s milk intake by an equal volume of formulated milk in young children (1–1.5 years) would improve vitamin D and iron adequacy [17]. A rapid increase in formulated milk consumption was observed in China from 2008 to 2013 [16], however, research on the role of formulated milk in the dietary intake of Chinese young children is very limited. Therefore, there is a need to assess the nutritional impacts of regular milk intake and formulated milk consumption in Chinese children.

The present study aims to investigate the potential nutritional impact of modelled dairy consumption in children aged 3–8 years, which includes two scenarios: one is adding an appropriate amount of cow’s milk or formulated milk powder for children aged 3 years and above (FMP3+) to their reported diet to meet the dairy intake recommendation, the other is substituting current milk consumption with FMP3+, using data from the China Health and Nutrition Survey (CHNS) in 2015.

## 2. Materials and Methods

### 2.1. Study Population

The data used in this study were from the 2015 CHNS, an ongoing and longitudinal study established in 1989 by the Chinese Center for Disease Control and Prevention, and the University of North Carolina at Chapel Hill. Participants were enrolled using a multistage stratified random sampling approach. Further details of the CHNS can be found in previous publications [23,24,25]. Compared to previous survey rounds [23,24], an additional three provinces were included in 2015 [25]. All subjects gave their informed consent for inclusion before they participated in the study. The study was conducted in accordance with the Declaration of Helsinki, and the protocol was approved by the Ethics Committee of National Institute for Nutrition and Health (NO. 201524).

**Figure 1:**
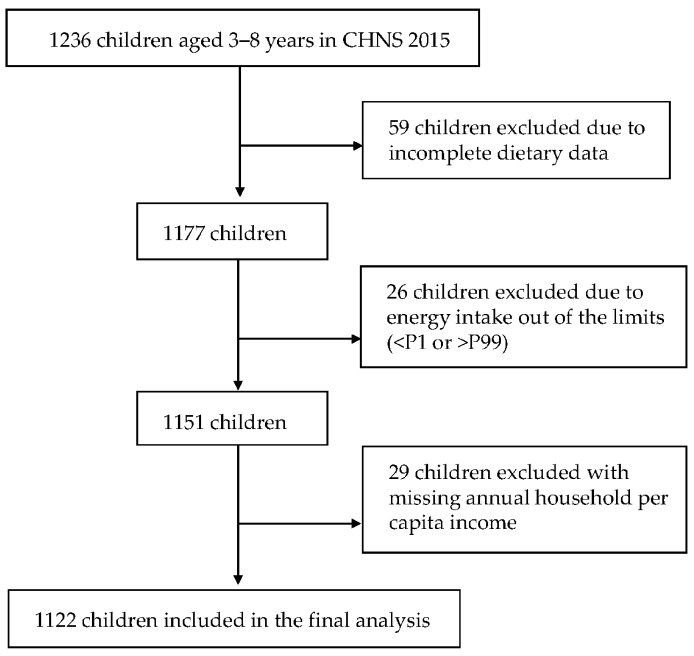
Sample flowchart of 3–8 year old participants in the China Health and Nutrition Survey 2015.

Children aged 3–8 years with completed data of socio-demographic characteristics and dietary measures were included in the present study. We excluded subjects with incomplete dietary data (*n* = 59) and implausible energy intake (<percentile (P) 1 or >P99, *n* = 26) [26,27], and those with missing data on annual household per capita income (*n* = 29). For participants with dietary intakes outside the sex-specific limits of the distribution (<P1 or >P99), we used corresponding sex-specific P1 and P99 to replace those of <P1 and >P99, respectively [26,27]. Finally, a total of 1122 children were included in the analysis (Figure 1).

### 2.2. Measurement of Dietary Intakes

Detailed dietary intake information for each household member aged 2 years and above over three consecutive days (two weekdays and one weekend day), including amount consumed, cooking method, and eating location, was recorded by a trained staff through a face-to-face interview using 24 hour dietary recalls. Briefly, mothers or caregivers who handled food preparation and feeding in the household were asked to recall the children’s food consumption over the past 24 h. Household edible oils, sugar, salt and sauce consumption were determined on a daily basis by calculating the changes in home food inventory by weighing and allocated to the child according to the proportion of energy intake in the family [10,27].

### 2.3. Estimation of Dairy Product Intakes

Food recalls were coded and analyzed to calculate dairy intakes according to the food categories in the Chinese Food Composition Table [28]. Dairy products were categorized into four sub-categories: fluid milk, milk powder, yogurt, and other dairy foods, which included cheese, condensed milk and milk tablets due to low consumption. The amounts of milk powder, yogurt and other dairy foods were converted into fluid milk equivalents based on their protein composition. In detail, the ratio of protein content per 100 g edible portion between each non-fluid dairy product and fluid milk was calculated and as a coefficient multiplied by the non-fluid dairy product consumption amount accordingly. The sum of fluid milk equivalents was counted as the total dairy intake in the study, and the percentage of children who did not meet the recommended 300 g/day of dairy intake by Dietary Guidelines for Chinese 2016 [11] was also calculated.

### 2.4. Estimation of Dietary Nutrient Intakes

The Chinese Food Composition Table [28] was used to estimate nutrient intakes. In the present study, daily intakes of energy and 18 nutrients were grouped into macronutrients (protein, fat, carbohydrate and dietary fiber), antioxidants (vitamins C and E), B vitamins (thiamin, riboflavin and niacin), bone-related nutrients (calcium, phosphorus and magnesium) and other micronutrients (vitamin A, iron, zinc, selenium, sodium and potassium). For each child, the total daily intake of each nutrient was estimated using the average of 3 days. The three energy contributors (protein, fat and carbohydrate) were also expressed as the percent of energy contribution (E%).

Estimated daily nutrient intakes were further evaluated against the Chinese Dietary Reference Intakes 2013 [29] when age-specific dietary intake recommendations were available. Prevalence of inadequate intakes were calculated as the proportion of children with average intake below the respective EAR of carbohydrate (g/day), protein (g/day), vitamin C (mg/day), thiamin (mg/day), riboflavin (mg/day), vitamin A (µg retinol activity equivalents (RAE)/day), calcium (mg/day), phosphorus (mg/day), magnesium (mg/day), iron (mg/day), zinc (mg/day), and selenium (µg/day) (Table S1). Adequate intake (AI) was used for evaluating sodium (mg/day), potassium (mg/day) and fat (E%, for children at 3–4 years) inadequacy, and the lower limit of AMDR was used to assess the inadequacy of carbohydrate (E%) and fat (E%, for children 4–8 years). Proposed intakes for preventing non-communicable chronic diseases (PI-NCD) of sodium was used to calculate the proportion of children with excessive sodium intake.

### 2.5. Measurement of Socio-Demographic Characteristics

Household and individual questionnaires were employed to collect information on household demographics and child characteristics. Data were stratified by child age (3–4 years and 5–8 years) and gender, mother’s educational level (categorized as low for primary school and below, mid for middle school, and high for high school and above), annual household income per capita (by tertiles) and residence region (urban and rural).

### 2.6. Modelling Scenarios

The present study modelled nutrient intakes in Chinese children using two scenarios. In scenario 1, we modelled the addition of the amount of FMP3+ or cow’s milk needed to bring each child’s dairy intake to the recommended amount, i.e., 300 g/day. Then, the modelled nutrient intakes and nutritional inadequacy were compared with actual reported intakes. In scenario 2, we modelled the substitution of “all milks” currently consumed in the reported diet with an equal volume of FMP3+. The nutritional impact of the substitution was assessed in comparison with current milk consumption. All types of milk and milk products, except plant-based milk and formulas, were within the definition of “all milks” in scenario 2, regardless of fat content, powder or liquid.

### 2.7. Statistical Analysis

Data analyses were performed using SAS 9.4 (SAS Inc., Cary, NC, USA). Continuous variables were presented as mean (standard deviation, SD) and percentiles, while categorical variables were expressed as a percent. The SAS procedure of “proc univariate normal” was used to test the normality of the distribution of continuous variables. Children were stratified by socio-demographic factors (age, gender, mother’s educational level, annual household income per capita and residence area). Differences in milk consumption for each demographic characteristic were compared by Chi-square test in the case of proportion, and otherwise Wilcoxon rank-sum test or Kruskal–Wallis H test, since the amounts of intakes are not normally distributed. Wilcoxon signed-rank test and paired Chi-square test were employed to test differences in the amounts of nutrients consumed and to estimate the proportions of children with nutrient inadequacy before and after modelling, respectively. A value of *p* < 0.05 was considered as statistically significant.

## 3. Results

### 3.1. Characteristics of Study Population and Their Dairy Consumption

The prevalence of dairy food consumption was 32.5% in our sample of 1122 Chinese children aged 3–8 years, with an average daily total dairy intake of 48.6 g (Table 1). Dairy intakes differed significantly by gender, mother’s education level, annual household income per capita, and residence area. Girls, children with high school and above educated mothers, and those from high-income families, as well as residents in urban areas, were found to consume more dairy foods compared to other groups (all *p* < 0.05). Overall, 97.6% children did not meet the recommended 300 g/day of dairy foods. 

Fluid milk, milk powder, yogurt and other dairy products were consumed by 25.1%, 1.8%, 11.0% and 0.5% of children, respectively (Table 2). Average intakes per capita were 30.9, 9.6, 9.0 and 0.3 g/day, respectively. Fluid milk consumption in girls (29.5%) was higher than boys (21.3%), and consequently, per capita consumption for girls (34.5 g/day) was also higher than for boys (27.7 g/day). Mothers with a high education level were more likely to feed children with fluid milk (40.1%) and yogurt (20.3%) relative to mothers with medium (19.0% and 6.3%, respectively) and low (17.3% and 6.8%, respectively) education levels. Both annual household income and residence area influenced fluid milk and yogurt consumption. A higher proportion of children living in urban areas consumed fluid milk and yogurt compared to rural children, and a higher proportion of younger children (3.9%) consumed milk powder than older children (0.6%) (Table 2).

### 3.2. Nutritional Impact of Meeting the Recommended Dairy Intake by Adding FMP3+ or Cow’s Milk

In Scenario 1 (Addition), either FMP3+ (model 1) or cow’s milk (model 2) was added to the diet of children who did not meet the dairy intake recommendation, to bring their intake to the recommended level, i.e., 300 g/day. As expected, significant changes in nutrient intakes and inadequacy status were observed (Table 3).

#### 3.2.1. Macronutrients

The median energy intake increased after modelling the addition of FMP3+ (model 1; 1363 kcal/day) or cow’s milk (model 2; 1327 kcal/day) as compared to reported consumption (1173 kcal/day). Similar increases in the amount of carbohydrate, protein and fat intake were found for each model. As a result, the proportions of children with inadequate intakes of carbohydrate and protein dropped in model 1 (19.2% and 6.2%, respectively) and model 2 (28.9% and 4.9%) compared to reported consumption (37.1% and 21.7%).

When the macronutrient intakes were evaluated by energy contribution (E%), the addition of cow’s milk in model 2 resulted in a lower energy contribution from carbohydrate (median 46.6%) compared to reported consumption (median 48.9%), whereas the addition of FMP3+ in model 1 showed the opposite (median 50.0 E%). The impact of models 1 and 2 on fat contribution to total energy was also different. Compared to reported consumption (median 37.2%), fat intakes were lower with the addition of FMP3+ (model 1; median 35.5%) whereas the addition of milk was higher (model 2; median 39.0%). Both models significantly increased the percentage of energy from protein, and it was significantly higher with the addition of cow’s milk compared to FMP3+. Dietary fiber was highest in model 1 with the addition of FMP3+ (median 7.6 g/day), compared to reported consumption (median 5.6 g/day) and the addition of cow’s milk model 2 (median 5.6 g/day).

#### 3.2.2. Antioxidant Vitamins

Compared to reported consumption (median 31.7 mg/day), vitamin C intake was significantly higher in model 1 (median 49.2 mg/day) and model 2 (median 34.4 mg/day). The proportion of children with inadequate intakes of vitamin C decreased by 45.9% after FMP3+ addition, while only a marginal decrease (3.0%) was observed after the addition of cow’s milk. Similar changes were also observed for vitamin E (α-TE) intake in model 1 and 2 relative to reported consumption.

#### 3.2.3. B-Vitamins

Compared to reported consumption, intakes of thiamin, riboflavin and niacin were significantly higher in both FMP3+ and cow’s milk addition models. Proportions of children with the risk of thiamin and riboflavin inadequacy were decreased in both models, and the improvement for thiamin was significantly higher in the FMP3+-added model 1, while that of riboflavin was significantly higher in the cow’s-milk-added model 2.

#### 3.2.4. Bone-Related Nutrients

Dietary calcium intakes after adding either FMP3+ or cow’s milk were about two times as high as in the reported diet, especially in added-cow’s-milk model 2. The intake of phosphorus and magnesium also increased. Accordingly, a decrease in the proportion of children with inadequate intakes of these nutrients was observed in both models.

#### 3.2.5. Other Micronutrients

FMP3+ addition (model 1) increased average vitamin A intake by 32.5% relative to the reported diet. Similar impact was found in the cow’s milk addition (model 2), although the improvement was less compared to model 1. A positive impact on mineral intakes was observed in both models, including iron, zinc, selenium, sodium, and potassium. By adding FMP3+ in model 1, the proportion of children with inadequate intakes of vitamin A decreased from 49.6% to 20.5%, and from 15.2% to 4.2% for iron, from 30.1% to 2.6% for zinc, from 75.0% to 39.8% for potassium, from 56.1% to 49.9% for selenium, and from 7.1% to 6.2% for sodium. However, the proportion of children with excessive sodium intake by comparison with PI-NCD was 87.5% in reported diet (data not shown), which was further increased in both model 1 (90.0%) and model 2 (89.8%).

### 3.3. Nutritional Impact of Substituting Milk Consumption by Equal Volume of FMP3+

The substitution scenario (Scenario 2) replaced current milk consumption with FMP3+ in both total children (model 3, *n* = 1122) and milk consumers (model 4, *n* = 291). All abovementioned nutrient intakes were evaluated, and the results are shown in Table 4.

#### 3.3.1. Macronutrients

In total children, relative to baseline diet, the replacement of consumed milk by the FMP3+ (model 3) marginally increased intakes of energy (median of model 3: 1190 kcal/day vs. baseline: 1173 kcal/day), and macronutrients such as carbohydrate (median: 146.5 g/day vs. 140.4 g/day) and protein (median: 37.1 g/day vs. 36.7 g/day), and a slight decrease in fat (median: 46.5 g/day vs. 46.6 g/day). Dietary fiber intake was found to increase significantly (median: 6.2 g/day vs. 5.6 g/day). In terms of energy contribution (E%), this slightly increased carbohydrate (median of model 3: 50.3% vs. reported consumption: 48.9%), whereas the E% of protein and fat was decreased after replacement.

All abovementioned changes in macronutrient intakes were observed in milk consumers when substituting milk consumption with an equal amount of FMP3+ (model 4).

#### 3.3.2. Antioxidant Vitamins

After the substitution, vitamin C intake was significantly increased in total children (median of model 3: 36.5 mg/day vs. baseline: 31.7 mg/day) and in milk consumers (median of model 4: 47.4 mg/day vs. baseline: 32.1 mg/day), resulting in a decrease in the proportion of children with inadequate vitamin C intake. Changes in the vitamin E intake were similar to that of vitamin C.

#### 3.3.3. B-Vitamins

The intakes of thiamin and niacin were higher after the substitution. Accordingly, the percentage of children with thiamin inadequate intake was decreased by 5.0% in total children and by 18.8% in milk consumers. Although the intake of riboflavin was similar, the percent of children below recommendation rose by 1.6% in total subjects and by 2.8% among milk consumers.

#### 3.3.4. Bone-Related Nutrients

Replacement of milk consumption by the FMP3+ in the diet of children induced improved intakes of bone-related nutrients in milk consumers, although the improvement was less pronounced in total children. As a result, the proportion of children with inadequate magnesium intake significantly decreased after the substitution in milk consumers, but no changes in the status of inadequate calcium and phosphorus intakes were found.

#### 3.3.5. Other Micronutrients

Vitamin A intake increased after the substitution in total children (median of model 3: 307.5 µg RAE/day vs. baseline: 278.5 µg RAE/day) and in milk consumers (median of model 4: 386.8 µg RAE/day vs. baseline: 307.4 µg RAE/day). A decrease in selenium intake was found. With the increased intakes of vitamin A, iron, zinc and potassium in models 3 and 4, the proportion of children with inadequate intakes of these nutrients improved in total children (model 3) as well as among milk consumers (model 4). The proportions of children with excessive sodium intake did not significantly change by the substitution of milk by equal volumes of FMP3+ relative to reported diet in both total children (87.7% vs. 87.5%) and milk consumers (90.0% vs. 89.4%, data not shown).

## 4. Discussion

Although the average percentage of consumption (from 2.2% in 1991 to 7.6% in 2006) and amount (from 14.9 g/day in 1992 to 24.7 g/day in 2012) of dairy foods consumed have increased in recent decades, dairy foods remain under-consumed in the Chinese population [13,14]. Very limited information is available on dairy consumption in Chinese young children. Zhang et al. [15] reported a lower consumption rate (14.8%) of milk and dairy products in rural Chinese children aged 4–17 years compared with those in highly (70.4%) and moderately (38.6%) urban areas in 2011, and daily energy contribution from dairy foods ranged from 6.0% to 7.8% on average. The findings in this study revealed the status of dairy food consumption and dietary nutrient intakes in Chinese children aged 3–8 years. A recent report of Chinese National Nutrition Survey 2010–2013 showed that the average total dairy and fluid milk intakes of 6–8 years old children was 39.5 g and 32.3 g per day, respectively [30], which are close to the 41.4 g/day (total dairy) and 31.4 g/day (fluid milk) found for 5–8 year old children in the present study.

Dairy food consumption is influenced by dietary culture and tradition, and unlike western countries [31], cheese is seldom consumed in China. Other factors also play a role, especially in developing countries, such as area of residence, mother’s education and wealth status of family [14,30,32,33]. In this study, we reported that over 97% 3–8 year old children did not meet the Chinese dairy intake recommendation of 300 g/day [11]. Similar low dairy consumption among preschool- and school-aged children has been reported in Asian countries including Indonesia and Vietnam [32,33]. The situation is better in western countries [34]. Higher daily dairy product intakes have also been reported for Australian children aged 2–3 years (416.3 g/day for girls and 434.4 g/day for boys) and 4–8 years (319.7 g/day for girls and 362.5 g/day for boys), and among Singapore children aged 3–6 and 7–10 years (635 g/day and 359 g/day, respectively) [31].

Our results highlighted high percentages of micronutrient inadequacies in Chinese children aged 3–8 years old, including vitamin C, thiamin, riboflavin, calcium, selenium and potassium, which were similar to the results of 4–17 year old children and adolescents using data from CHNS 2011 [10]. Significant inadequate intakes of thiamin and calcium were also reported in 2–6 year old Chinese children from CHNS 2011 [35]. Low dairy food consumption could be one of the reasons for the high nutrient intake inadequacy in Chinese children [14]. Indeed, dairy foods are important dietary sources of multiple micronutrients, including calcium, phosphorous, magnesium, zinc, potassium, vitamin A, vitamin D, riboflavin and vitamin B12 [13,31]. In addition, dairy products provide children with energy, high-quality protein, and essential and nonessential fatty acids. Dairy foods are an essential component of a nutrient-rich and balanced diet. Therefore, increasing the consumption of dairy products could improve the nutritional status of Chinese children. However, Chinese diets are traditionally high in plant foods; interventions to promote dairy intake are needed. Considering the decreased frequency of dairy intake among Chinese population with ageing [13], and the disparities of dairy consumption between urban and rural areas, as well as among different income levels [13,14], targeted intervention strategies such as dietary and nutritional education according to Dietary Guidelines for Chinese by health workers, a free/discounted dairy food supply for young children and elders, or affordable dairy consumption in general population by government subsidy could be encouraged. On the other hand, since many Asians are lactose intolerant, diverse dairy foods should be produced, including lactose-free dairy products. In the Dietary Guidelines for Chinese [11], a large daily amount of fruit, vegetable and dairy intakes was recommended, and a proper amount for fish or shellfish, eggs, meat and poultry. Excessive meat consumption leads to high intakes of saturated fat, cholesterol and other substances associated with negative impacts on health. Meat consumption has also been identified as a dietary risk factor for coronary heart disease, obesity, diabetes, colorectal cancer and stroke [13]. Therefore, it is recommended to increase dairy intake to meet recommendation of 300 g/day by displacing excessive meat consumption in Chinese. If so, the nutrient status would be better for human heath with lower inadequacy, especially for calcium, phosphorous, magnesium, potassium and vitamin D, and less dietary intakes of saturated fat and cholesterol.

To study if and to what extend increased dairy food consumption could improve the nutritional condition of Chinese children, we modeled the addition of FMP3+ or milk in this study. Diet modelling methods have been used in other studies of dairy intakes. Using data from the US National Health and Nutrition Examination Survey, diet modelling studies showed that increasing dairy intakes would decrease the percent of children with inadequate calcium intakes in 4–8 year olds [20] and 2–18 year olds [21]. In our study, achieving the daily recommended consumption of dairy products by adding either the FMP3+ or cow’s milk (Scenario 1) improved nutrient intakes and brought more children in accordance with the recommendations. However, this approach increased energy intakes, which also needs to be considered. In Scenario 2, FMP3+ was used to substitute existing milk consumption with slight impact on energy intakes, and improved intakes of vitamins A and C, thiamin, iron, zinc and potassium, which means that FMP3+ provides further improvement compared to currently consumed milk. However, this approach does not bring children to the recommended dairy intake level.

There are limitations of the present study that should be considered when interpreting the results. First, milk in Scenario 2 was defined as all milk types except plant-based milk and formulas, regardless of fat content, powder or liquid. A larger sample size would be needed to evaluate more complex simulation scenarios taking into account different types of milk and diverse dairy consumption patterns. Second, in the present study, the average of 3 days of nutrient intakes were used in the analysis, which may overestimate the prevalence of inadequate intakes compared to usual nutrient intake estimations [36]. Third, the contribution of dietary supplements was not considered in the present study because the related consumption data were not collected. However, the effect of dietary supplements on nutrient intakes is likely to be small in this population because the use of dietary supplements in Chinese children is much lower than other countries [10]. Additionally, the intakes of vitamin D and different types of fatty acids were not assessed in the present study due to the lack of information in the China food composition table.

## 5. Conclusions

In conclusion, both the percentage and amount of dairy consumption in Chinese children aged 3–8 years are at a quite low level, especially among older children and those from rural households. The poor dairy intake is partly reflected by a high prevalence of inadequate intakes for many nutrients. Increasing dairy consumption, either FMP3+ or cow’s milk, is an approach that could improve the overall diet quality of Chinese children. Taken together, strategies to promote a healthy varied diet and increase the consumption of fortified foods are still needed for optimal nutritional intakes.

## Figures and Tables

**Table 1:** Total dairy equivalent consumption (g/day) by demographic characteristics of 3–8 year old participants in the China Health and Nutrition Survey 2015 ^a^.

Characteristics	*N* (%)	Total Dairy							% Below Dairy Recommendation	*p*-Value ^b^
		Percent (%)	*p*-Value ^b^	Mean (SD)	P50 (P25, P75)	P90	P95 ^c^	*p*-Value ^d^		
Age group			0.3353					0.3347		0.0036
3–4 years	408 (36.4)	34.3		61.1 (151.6)	0.0 (0.0, 66.7)	200.0	250.0		95.8	
5–8 years	714 (63.6)	31.5		41.4 (76.4)	0.0 (0.0, 66.7)	153.3	211.1		98.6	
Gender			**0.0002**					**0.0024**		0.5175
boys	596 (53.1)	27.7		46.6 (118.6)	0.0 (0.0, 50.0)	166.7	237.2		97.3	
girls	526 (46.9)	38.0		50.8 (100.0)	0.0 (0.0, 70.0)	166.7	208.3		97.9	
Mother’s education level			**< 0.0001**					**< 0.0001**		0.3434
low	411 (36.6)	22.6		42.3 (124.0)	0.0 (0.0, 0.0)	166.7	250.0		97.1	
mid	352 (31.4)	23.3		33.3 (88.9)	0.0 (0.0, 0.0)	116.7	211.1		98.6	
high	359 (32.0)	52.9		70.8 (109.3)	33.3 (0.0, 111.1)	194.4	250.0		97.2	
Annual household income level			**< 0.0001**					**< 0.0001**		0.7106
low	373 (33.2)	19.8		39.2 (132.2)	0.0 (0.0, 0.0)	133.3	237.2		97.3	
mid	375 (33.4)	30.9		42.6 (100.5)	0.0 (0.0, 50.0)	140.0	219.4		97.3	
high	374 (33.3)	46.8		63.9 (92.7)	0.0 (0.0, 93.3)	200.0	250.0		98.1	
Residence area			**< 0.0001**					**< 0.0001**		0.0012
urban	346 (30.8)	53.8		88.0 (141.6)	40.8 (0.0, 133.3)	233.3	285.6		95.4	
rural	776 (69.2)	23.1		31.0 (87.5)	0.0 (0.0, 0.0)	100.0	166.7		98.6	
Total	1122 (100)	32.5		48.6 (110.2)	0.0 (0.0, 66.7)	166.7	233.3		97.6	

Abbreviation: SD, standard deviation. ^a^ Total dairy intake is the sum of fluid milk equivalents, including fluid milk, milk powder, yogurt and other dairy foods. ^b^ Chi-square test comparing the percentages of children who consumed dairy food, and the proportions of children not meeting the recommended 300 g/day of dairy intake. ^c^ P25, 50, 75, 90 and 95 are percentiles of total dairy intake. The lower percentiles are not shown since the consumption is zero. ^d^ Wilcoxon rank sum test or Kruskal–Wallis H test was performed to compare intake.

**Table 2:** Consumption of fluid milk equivalents (g/day) by demographic characteristics of 3–8 year old participants in the China Health and Nutrition Survey 2015 (*n* = 1122) ^a^.

Characteristics	Fluid Milk Equivalent						
	*N* (%)	*p*-Value ^b^	Mean (SD)	P50 (P25, P75)	P90	P95^c^	*p*-Value ^d^
**Fluid milk**							
Age group		0.9481					0.8334
3–4 years	103 (25.3)		30.0 (67.8)	0.0 (0.0, 29.5)	125.0	166.7	
5–8 years	179 (25.1)		31.4 (64.6)	0.0 (0.0, 26.7)	133.3	166.7	
Gender		**0.0017**					**0.0034**
boys	127 (21.3)		27.7 (66.8)	0.0 (0.0, 0.0)	116.7	166.7	
girls	155 (29.5)		34.5 (64.4)	0.0 (0.0, 60.0)	140.0	166.7	
Mother’s education level		**< 0.0001**					**< 0.0001**
low	71 (17.3)		26.0 (67.4)	0.0 (0.0, 0.0)	116.7	200.0	
mid	67 (19.0)		21.0 (51.1)	0.0 (0.0, 0.0)	83.3	141.7	
high	144 (40.1)		46.2 (73.6)	0.0 (0.0, 83.3)	153.3	200.0	
Annual household income		**< 0.0001**					**< 0.0001**
low	52 (13.9)		18.7 (56.0)	0.0 (0.0, 0.0)	76.9	150.0	
mid	82 (21.9)		27.0 (67.1)	0.0 (0.0, 0.0)	100.0	166.7	
high	148 (39.6)		46.9 (70.2)	0.0 (0.0, 83.3)	166.7	200.0	
Residence area		**< 0.0001**					**< 0.0001**
urban	147 (42.5)		53.6 (77.9)	0.0 (0.0, 83.3)	166.7	210.0	
rural	135 (17.4)		20.8 (56.8)	0.0 (0.0, 0.0)	83.3	153.3	
Total	282 (25.1)		30.9 (65.8)	0.0 (0.0, 26.7)	133.3	166.7	
**Milk powder**							
Age group		**< 0.0001**					**< 0.0001**
3–4 years	16 (3.9)		24.0 (178.8)	0.0 (0.0, 0.0)	0.0	0.0	
5–8 years	4 (0.6)		1.3 (20.2)	0.0 (0.0, 0.0)	0.0	0.0	
Gender		0.5338					0.5328
boys	12 (2.0)		12.3 (136.7)	0.0 (0.0, 0.0)	0.0	0.0	
girls	8 (1.5)		6.4 (66.3)	0.0 (0.0, 0.0)	0.0	0.0	
Mother’s education level		0.5257					0.5256
low	9 (2.2)		14.5 (160.3)	0.0 (0.0, 0.0)	0.0	0.0	
mid	4 (1.1)		5.1 (60.1)	0.0 (0.0, 0.0)	0.0	0.0	
high	7 (2.0)		8.2 (67.2)	0.0 (0.0, 0.0)	0.0	0.0	
Annual household income		0.2033					0.2021
low	9 (2.4)		19.4 (177.3)	0.0 (0.0, 0.0)	0.0	0.0	
mid	3 (0.8)		3.3 (49.4)	0.0 (0.0, 0.0)	0.0	0.0	
high	8 (2.1)		6.0 (45.7)	0.0 (0.0, 0.0)	0.0	0.0	
Residence area		**0.0001**					**< 0.0001**
urban	14 (4.1)		22.0 (177.4)	0.0 (0.0, 0.0)	0.0	0.0	
rural	6 (0.8)		4.0 (56.7)	0.0 (0.0, 0.0)	0.0	0.0	
Total	20 (1.8)		9.6 (109.5)	0.0 (0.0, 0.0)	0.0	0.0	
**Yogurt**							
Age group		**0.1485**					**0.1719**
3–4 years	52 (12.8)		10.0 (34.6)	0.0 (0.0, 0.0)	41.7	69.4	
5–8 years	71 (9.9)		8.4 (31.8)	0.0 (0.0, 0.0)	0.0	69.4	
Gender		0.5228					0.5379
boys	62 (10.4)		8.4 (30.2)	0.0 (0.0, 0.0)	27.8	66.7	
girls	61 (11.6)		9.6 (35.7)	0.0 (0.0, 0.0)	27.8	69.4	
Mother’s education level		< 0.0001					< 0.0001
low	28 (6.8)		5.0 (20.6)	0.0 (0.0, 0.0)	0.0	47.2	
mid	22 (6.3)		7.2 (38.5)	0.0 (0.0, 0.0)	0.0	46.3	
high	73 (20.3)		15.2 (37.2)	0.0 (0.0, 0.0)	66.7	97.2	
Annual household income		**0.0005**					**0.0006**
low	23 (6.2)		5.2 (25.3)	0.0 (0.0, 0.0)	0.0	48.1	
mid	44 (11.7)		11.4 (42.2)	0.0 (0.0, 0.0)	27.8	94.4	
high	56 (15.0)		10.3 (28.2)	0.0 (0.0, 0.0)	55.6	81.5	
Residence area		**< 0.0001**					**< 0.0001**
urban	71 (20.5)		15.7 (37.5)	0.0 (0.0, 0.0)	69.4	97.2	
rural	52 (6.7)		6.0 (30.1)	0.0 (0.0, 0.0)	0.0	44.4	
Total	123 (11.0)		9.0 (32.8)	0.0 (0.0, 0.0)	27.8	69.4	
**Other dairy**							
Age group		0.4256					0.3172
3–4 years	1 (0.3)		0.4 (7.8)	0.0 (0.0, 0.0)	0.0	0.0	
5–8 years	5 (0.7)		0.3 (4.5)	0.0 (0.0, 0.0)	0.0	0.0	
Gender		1.0000					0.8815
boys	3 (0.5)		0.5 (7.9)	0.0 (0.0, 0.0)	0.0	0.0	
girls	3 (0.6)		0.1 (2.1)	0.0 (0.0, 0.0)	0.0	0.0	
Mother’s education level		0.1996					0.1849
low	4 (1.0)		0.4 (5.9)	0.0 (0.0, 0.0)	0.0	0.0	
mid	0 (0.0)		0.0 (0.0)	0.0 (0.0, 0.0)	0.0	0.0	
high	2 (0.6)		0.5 (8.4)	0.0 (0.0, 0.0)	0.0	0.0	
Annual household income		0.1755					0.1363
low	0 (0.0)		0.0 (0.0)	0.0 (0.0, 0.0)	0.0	0.0	
mid	4 (1.1)		0.4 (5.9)	0.0 (0.0, 0.0)	0.0	0.0	
high	2 (0.5)		0.5 (8.4)	0.0 (0.0, 0.0)	0.0	0.0	
Residence area		0.0768					0.0566
urban	4 (1.2)		0.9 (10.5)	0.0 (0.0, 0.0)	0.0	0.0	
rural	2 (0.3)		0.05 (1.1)	0.0 (0.0, 0.0)	0.0	0.0	
Total	6 (0.5)		0.3 (5.9)	0.0 (0.0, 0.0)	0.0	0.0	

Abbreviation: SD, standard deviation. ^a^ Intakes of milk powder, yogurt and other dairy were converted to that of fluid milk based on protein content. Other dairy includes cheese, butter, condensed milk and milk tablets. ^b^ Chi-square test comparing the percentages of children who consumed respective dairy food. ^c^ P25, 50, 75, 90 and 95 are percentiles of each dairy food. The lower percentiles are not shown since the consumption is zero. ^d^ Wilcoxon rank sum test or Kruskal–Wallis H test was performed to compare intakes.

**Table 3:** Daily nutrient intakes of children aged 3–8 years, comparing reported intakes with the addition of FMP3+ (model 1) and milk (model 2).

Nutrients	Groups	Dietary Nutrient Intake						Children With Inadequate Intake
		Mean ± SD	P10	P25	Median	P75	P90	*N* (%)
Energy (kcal/day)	reported-T ^a^	1252 ± 498	672	886	1173	1544	1926	NA
	model1 ^b^	1439 ± 497	861	1077	1363	1730	2108	NA
	model2 ^b,c^	1398 ± 497	813	1033	1327	1680	2068	NA
Carbohydrate (g/day)	reported-T	155.6 ± 74.4	77.5	105.6	140.4	190.2	247.9	416 (37.1)
	model1 ^b^	180.8 ± 74.0	102.1	130.6	165.5	216.3	274.5	215 (19.2) ^d^
	model2 ^b,c^	164.8 ± 74.1	86.0	115.1	149.9	199.9	258.1	324 (28.9) ^d,e^
Carbohydrate (E%)	reported-T	49.6 ± 12.3	34.2	41.0	48.9	57.9	66.6	596 (53.1)
	model1 ^b^	50.5 ± 10.5	37.0	43.4	50.0	57.6	64.9	558 (49.7) ^d^
	model2 ^b,c^	46.9 ± 10.7	33.5	39.4	46.6	53.9	61.1	684 (61.0) ^d,e^
Protein (g/day)	reported-T	39.5 ± 17.3	19.5	26.3	36.7	50.0	62.6	243 (21.7)
	model1 ^b^	47.7 ± 17.3	27.7	34.6	44.7	58.3	70.7	69 (6.2) ^d^
	model2 ^b,c^	47.6 ± 17.0	28.0	34.8	44.3	58.4	70.4	55 (4.9) ^d,e^
Protein (E%)	reported-T	13.0 ± 3.2	9.4	10.9	12.7	14.8	17.3	NA
	model1 ^b^	13.5 ± 2.7	10.5	11.7	13.2	15.0	17.0	NA
	model2 ^b,c^	13.9 ± 2.7	10.8	12.1	13.6	15.5	17.6	NA
Fat (g/day)	reported-T	51.8 ± 27.1	21.3	31.3	46.6	66.5	88.7	NA
	model1 ^b^	57.2 ± 27.0	26.6	36.5	52.3	72.3	94.2	NA
	model2 ^b,c^	60.5 ± 26.9	30.1	40.3	55.4	75.3	96.9	NA
Fat (E%)	reported-T	37.2 ± 12.0	21.8	29.4	37.2	44.9	52.9	145 (12.9)
	model1 ^b^	35.6 ± 10.3	22.2	28.8	35.5	42.6	49.0	151 (13.5)
	model2 ^b,c^	39.2 ± 10.5	25.8	32.0	39.0	46.0	52.8	95 (8.5) ^d,e^
Dietary fiber (g/day)	reported-T	6.6 ± 4.2	2.7	3.8	5.6	8.3	11.1	NA
	model1 ^b^	8.6 ± 4.3	4.5	5.7	7.6	10.2	13.1	NA
	model2 ^c^	6.6 ± 4.2	2.7	3.8	5.6	8.3	11.1	NA
Vitamin C (mg/day)	reported-T	40.4 ± 30.4	10.9	19.8	31.7	53.0	78.7	719 (64.1)
	model1 ^b^	57.5 ± 30.1	27.6	36.2	49.2	70.3	96.4	389 (34.7)^d^
	model2 ^b,c^	43.1 ± 30.4	13.6	22.5	34.4	55.6	81.7	685 (61.1) ^d,e^
Vitamin E (α-TE, mg/day)	reported-T	3.7 ± 2.4	1.1	1.9	3.0	4.9	7.1	NA
	model1 ^b^	5.0 ± 2.4	2.5	3.3	4.4	6.3	8.4	NA
	model2 ^b,c^	3.9 ± 2.4	1.4	2.2	3.3	5.2	7.3	NA
Thiamin (mg/day)	reported-T	0.5 ± 0.3	0.2	0.3	0.5	0.7	0.9	792 (70.6)
	model1 ^b^	0.7 ± 0.3	0.4	0.5	0.6	0.8	1.0	557 (49.6) ^d^
	model2 ^b,c^	0.6 ± 0.3	0.3	0.4	0.6	0.7	1.0	679 (60.5) ^d,e^
Riboflavin (mg/day)	reported-T	0.5 ± 0.3	0.2	0.3	0.5	0.7	0.9	761 (67.8)
	model1 ^b^	0.8 ± 0.3	0.5	0.6	0.7	0.9	1.2	350 (31.2) ^d^
	model2 ^b,c^	0.9 ± 0.3	0.7	0.7	0.9	1.1	1.3	87 (7.8) ^d,e^
Niacin (mg/day)	reported-T	8.8 ± 4.3	4.1	5.7	8.0	11.5	14.6	NA
	model1 ^b^	10.1 ± 4.3	5.3	7.0	9.3	12.7	15.8	NA
	model2 ^b,c^	9.1 ± 4.3	4.3	6.0	8.3	11.7	14.8	NA
Calcium (mg/day)	reported-T	256.6 ± 170.9	94.8	143.1	209.9	331.7	464.2	1089 (97.1)
	model1 ^b^	470.3 ± 165.1	306.4	353.6	422.8	554.9	690.0	960 (85.6) ^d^
	model2 ^b,c^	539.3 ± 151.4	397.9	440.8	500.5	591.1	727.2	918 (81.8) ^d,e^
Phosphorus (mg/day)	reported-T	580.5 ± 245.6	303.2	393.4	535.9	723.0	910.3	105 (9.4)
	model1 ^b^	762.3 ± 243.2	485.5	575.3	720.5	905.0	1095.2	1 (0.1) ^d^
	model2 ^b,c^	778.6 ± 238.9	506.8	597.8	735.0	916.2	1096.0	0 (0.0)
Magnesium (mg/day)	reported-T	158.9 ± 73.8	79.5	105.9	143.7	196.0	251.7	518 (46.2)
	model1 ^b^	183.3 ± 74.1	104.3	129.7	167.8	220.3	274.8	305 (27.2) ^d^
	model2 ^b,c^	188.8 ± 73.5	109.1	136.4	172.7	225.7	282.8	272 (24.2) ^d,e^
Vitamin A (µg RAE/day)	reported-T	414.6 ± 466.2	87.9	144.9	278.5	489.7	887.6	557 (49.6)
	model1 ^b^	549.4 ± 466.5	223.9	280.3	409.1	624.5	1015.7	230 (20.5) ^d^
	model2 ^b,c^	479.8 ± 466.5	153.1	210.5	338.8	553.7	945.1	420(37.4) ^d,e^
Iron (mg/day)	reported-T	13.2 ± 7.4	6.3	8.4	11.6	15.9	21.5	171 (15.2)
	model1 ^b^	15.3 ± 7.4	8.4	10.5	13.6	18.0	23.7	47 (4.2) ^d^
	model2 ^b,c^	14.0 ± 7.4	7.2	9.2	12.4	16.7	22.4	113 (10.1) ^d,e^
Zinc (mg/day)	reported-T	6.3 ± 2.8	3.1	4.3	5.8	7.8	10.1	338 (30.1)
	model1 ^b^	8.8 ± 2.7	5.7	6.8	8.3	10.4	12.6	29 (2.6) ^d^
	model2 ^b,c^	7.4 ± 2.7	4.3	5.4	6.9	9.0	11.2	130 (11.6) ^d,e^
Selenium (µg/day)	reported-T	27.3 ± 14.4	11.8	17.0	24.1	34.0	46.4	629 (56.1)
	model1 ^b^	29.0 ± 14.5	13.5	18.6	26.1	35.8	48.3	560 (49.9) ^d^
	model2 ^b,c^	32.5 ± 14.2	17.4	22.2	29.5	39.5	50.8	412 (36.7) ^d,e^
Sodium (mg/day)	reported-T	2916.1 ± 1980.0	1062.1	1622.1	2448.8	3586.5	5154.9	80 (7.1)
	model1 ^b^	3035.0 ± 2002.0	1177.5	1735.7	2564.2	3701.9	5256.5	70 (6.2) ^d^
	model2 ^b,c^	3017.1 ± 1979.0	1164.6	1719.2	2547.7	3680.6	5243.3	71 (6.3) ^d^
Potassium (mg/day)	reported-T	986.4 ± 468.3	460.2	643.2	907.5	1238.9	1586.1	841 (75.0)
	model1 ^b^	1378.9 ± 463.0	862.0	1030.8	1298.1	1627.3	1956.5	447 (39.8) ^d^
	model2 ^b,c^	1282.6 ± 461.3	773.4	944.9	1202.1	1514.5	1866.5	567 (50.5) ^d,e^

Models 1 and 2 added FMP3+ and cow’s milk, respectively, to the diets of children not meeting dairy recommendation to bring them to the recommended level of 300g/day. Abbreviation: SD, standard deviation; NA, no Chinese EAR available. ^a^ reported-T: reported intakes in total children (*n* = 1122). Wilcoxon signed-rank test was used to compare the intakes, ^b^ *p* < 0.01 when comparing reported-T intakes with each modelled intake, ^c^ *p* < 0.01 when comparing model 1 and 2. Paired Chi-square test was employed to compare the proportions of children below nutrient intake recommendations, ^d^ *p* < 0.001 when comparing reported-T with each model, and ^e^ *p* < 0.001 when model 1 and 2 were compared.

**Table 4:** Daily nutrient intakes of children aged 3–8 years, comparing reported intakes with the modelling where milk consumption was substituted by equal volume of FMP3+.

Nutrients	Groups	Dietary Nutrient Intake						Children With Inadequate Intake
		Mean ± SD	P10	P25	P50	P75	P90	*N* (%)
Energy (kcal/day)	reported-T	1252 ± 498	672	886	1173	1544	1926	NA
	model3 ^a^	1270 ± 501	687	903	1190	1557	1946	NA
	reported-M	1321 ± 462	734	973	1291	1605	1880	NA
	model 4 ^a^	1387 ± 459	805	1059	1375	1658	1947	NA
Carbohydrate (g/day)	reported-T	155.6 ± 74.1	77.5	105.6	140.4	190.2	247.9	416 (37.1)
	model3 ^a^	160.0 ± 74.8	80.7	108.8	146.5	198.2	254.1	378 (33.7) ^b^
	reported-M	156.8 ± 68.3	80.3	110.1	144.5	189.9	242.1	98 (33.7)
	model 4 ^a^	171.9 ± 68.1	96.8	124.3	157.5	205.2	263.7	63 (21.7) ^b^
Carbohydrate (E%)	reported-T	49.6 ± 12.3	34.2	41.0	48.9	57.9	66.6	596 (53.1)
	model3 ^a^	50.6 ± 12.0	35.3	42.5	50.3	58.4	66.7	553 (49.3) ^b^
	reported-M	47.0 ± 10.3	33.9	39.6	46.5	53.9	59.7	185 (63.6)
	model 4 ^a^	49.7 ± 9.7	36.7	42.8	49.5	55.9	61.8	157 (54.0) ^b^
Protein (g/day)	reported-T	39.5 ± 17.3	19.5	26.3	36.7	50.0	62.6	243 (21.7)
	model3 ^a^	40.0 ± 17.6	19.7	26.7	37.1	50.7	64.2	236 (21.0) ^b^
	reported-M	44.6 ± 17.3	24.2	32.3	41.3	56.2	67.8	34 (11.7)
	model 4 ^a^	46.7 ± 17.5	25.8	34.5	44.0	58.6	70.6	27 (9.3) ^b^
Protein (E%)	reported-T	13.0 ± 3.2	9.4	10.9	12.7	14.8	17.3	NA
	model3 ^a^	12.7 ± 3.0	9.3	10.7	12.4	14.5	16.9	NA
	reported-M	14.0 ± 3.2	10.2	11.7	13.5	15.9	18.4	NA
	model 4 ^a^	13.6 ± 3.0	10.1	11.7	13.2	15.3	17.6	NA
Fat (g/day)	reported-T	51.8 ± 27.1	21.3	31.3	46.6	66.5	88.7	NA
	model3 ^a^	51.5 ± 26.8	21.1	31.0	46.5	66.1	87.6	NA
	reported-M	57.0 ± 25.5	27.8	39.2	53.0	71.9	93.5	NA
	model 4	56.2 ± 24.9	27.2	38.4	53.0	69.5	92.1	NA
Fat (E%)	reported-T	37.2 ± 12.0	21.8	29.4	37.2	44.9	52.9	145 (12.9)
	model3 ^a^	36.4 ± 11.9	21.2	28.8	36.2	44.1	52.0	154 (13.7) ^b^
	reported-M	38.8 ± 10.1	26.7	32.3	38.8	45.4	51.5	28 (9.6)
	model 4 ^a^	36.2 ± 9.7	24.1	30.3	35.9	42.2	48.6	36 (12.4) ^b^
Dietary fiber (g/day)	reported-T	6.6 ± 4.2	2.7	3.8	5.6	8.3	11.1	NA
	model3 ^a^	7.2 ± 4.4	3.0	4.2	6.2	8.9	12.1	NA
	reported-M	6.6 ± 3.9	2.6	4.0	5.8	8.6	10.8	NA
	model 4 ^a^	8.3 ± 4.1	4.3	5.3	7.3	10.4	13.1	NA
Vitamin C (mg/day)	reported-T	40.4 ± 30.4	10.9	19.8	31.7	53.0	78.7	719 (64.1)
	model3 ^a^	44.2 ± 30.8	13.4	23.2	36.5	57.1	84.1	653 (58.2) ^b^
	reported-M	40.4 ± 29.2	11.2	20.1	32.1	53.6	76.8	178 (61.2)
	model 4 ^a^	53.9 ± 29.6	22.7	33.5	47.4	70.0	94.3	116 (39.9) ^b^
Vitamin E (α-TE, mg/day)	reported-T	3.7 ± 2.4	1.1	1.9	3.0	4.9	7.1	NA
	model3 ^a^	4.0 ± 2.5	1.3	2.0	3.3	5.2	7.6	NA
	reported-M	3.8 ± 2.3	1.3	2.0	3.3	5.3	7.1	NA
	model 4 ^a^	4.9 ± 2.5	2.1	3.1	4.3	6.4	8.1	NA
Thiamin (mg/day)	reported-T	0.5 ± 0.3	0.2	0.3	0.5	0.7	0.9	792 (70.6)
	model3 ^a^	0.6 ± 0.3	0.3	0.4	0.5	0.7	0.9	753 (67.1) ^b^
	reported-M	0.6 ± 0.2	0.3	0.4	0.5	0.7	0.9	191 (65.6)
	model 4 ^a^	0.6 ± 0.3	0.3	0.5	0.6	0.8	1.0	155 (53.3) ^b^
Riboflavin (mg/day)	reported-T	0.5 ± 0.3	0.2	0.3	0.5	0.7	0.9	761 (67.8)
	model3	0.5 ± 0.3	0.2	0.3	0.5	0.7	0.9	773 (68.9) ^b^
	reported-M	0.7 ± 0.3	0.4	0.5	0.7	0.9	1.0	115 (39.5)
	model 4 ^a^	0.7 ± 0.3	0.4	0.5	0.7	0.9	1.0	118 (40.6)
Niacin (mg/day)	reported-T	8.8 ± 4.3	4.1	5.7	8.0	11.5	14.6	NA
	model3 ^a^	9.1 ± 4.3	4.2	6.0	8.4	11.8	15.0	NA
	reported-M	9.4 ± 4.2	4.7	6.4	8.6	11.9	14.9	NA
	model 4 ^a^	10.4 ± 4.3	5.7	7.3	9.4	12.7	16.3	NA
Calcium (mg/day)	reported-T	256.6 ± 170.9	94.8	143.1	209.9	331.7	464.2	1089 (97.1)
	model3 ^a^	256.7 ± 161.4	94.8	141.4	212.2	336.8	476.3	1090 (97.2)
	reported-M	370.2 ± 182.2	189.0	255.3	342.6	441.3	574.3	270 (92.8)
	model 4 ^a^	383.6 ± 161.3	205.1	261.8	364.2	476.3	593.2	269 (92.4)
Phosphorus (mg/day)	reported-T	580.5 ± 245.6	303.2	393.4	535.9	723.0	910.3	105 (9.4)
	model3 ^a^	587.9 ± 247.2	305.2	399.2	546.7	736.1	928.7	103 (9.2)
	reported-M	665.4 ± 237.9	378.0	494.3	643.6	812.5	994.9	5 (1.7)
	model 4 ^a^	699.1 ± 237.0	414.9	520.6	686.0	852.1	1027.5	2 (0.7)
Magnesium (mg/day)	reported-T	158.9 ± 73.8	79.5	105.9	143.7	196.0	251.7	518 (46.2)
	model3 ^a^	159.6 ± 74.0	80.4	106.3	145.0	196.6	251.3	509 (45.4) ^b^
	reported-M	169.4 ± 70.6	91.1	119.3	157.8	207.4	248.4	105 (36.1)
	model 4 ^a^	171.8 ± 69.5	96.5	120.4	160.7	209.5	247.2	96 (33.0) ^b^
Vitamin A (µg RAE/day)	reported-T	414.6 ± 466.2	87.9	144.9	278.5	489.7	887.6	557 (49.6)
	model3 ^a^	435.8 ± 468.5	90.4	166.0	307.5	519.1	917.7	509 (45.4) ^b^
	reported-M	407.8 ± 384.2	112.5	178.7	307.4	497.3	768.8	123 (42.3)
	model 4 ^a^	486.4 ± 389.8	171.1	256.3	386.8	556.8	888.2	79 (27.2) ^b^
Iron (mg/day)	reported-T	13.2 ± 7.4	6.3	8.4	11.6	15.9	21.5	171 (15.2)
	model3 ^a^	13.6 ± 7.4	6.6	8.7	12.1	16.2	22.1	153 (13.6) ^b^
	reported-M	13.7 ± 7.5	7.0	8.9	12.2	16.3	21.6	32 (11.0)
	model 4 ^a^	15.1 ± 7.5	7.9	10.1	13.4	17.6	23.8	17 (5.8) ^b^
Zinc (mg/day)	reported-T	6.3 ± 2.8	3.1	4.3	5.8	7.8	10.1	338 (30.1)
	model3 ^a^	6.7 ± 3.0	3.2	4.5	6.2	8.6	10.7	288 (25.7) ^b^
	reported-M	6.8 ± 2.6	3.8	4.9	6.5	8.3	10.3	62 (21.3)
	model 4 ^a^	8.3 ± 2.8	5.0	6.1	7.9	10.1	12.2	17 (5.8) ^b^
Selenium (µg/day)	reported-T	27.3 ± 14.4	11.8	17.0	24.1	34.0	46.4	629 (56.1)
	model3 ^a^	26.7 ± 14.2	11.6	16.5	23.4	33.3	45.4	650 (57.9) ^b^
	reported-M	31.3 ± 15.1	15.4	19.9	28.3	40.9	53.5	124 (42.6)
	model 4 ^a^	29.5 ± 15.0	13.3	18.1	27.0	38.5	51.2	141 (48.5) ^b^
Sodium (mg/day)	reported-T	2916.1 ± 1980.0	1062.1	1622.1	2448.8	3586.5	5154.9	80 (7.1)
	model3 ^a^	2931.6 ± 2003.0	1074.4	1629.3	2450.5	3612.8	5151.5	77 (6.9)
	reported-M	2973.1 ± 1829.0	1093.6	1689.2	2585.0	3764.5	5184.2	22 (7.6)
	model 4 ^a^	3012.2 ± 1844.0	1113.7	1735.5	2612.0	3834.5	5250.5	19 (6.5)
Potassium (mg/day)	reported-T	986.4 ± 468.3	460.2	643.2	907.5	1238.9	1586.1	841 (75.0)
	model3 ^a^	1035.0 ± 505.1	475.5	670.9	944.1	1310.7	1706.3	792 (70.6) ^b^
	reported-M	1129.1 ± 451.1	614.6	816.1	1070.1	1340.5	1739.5	179 (61.5)
	model 4 ^a^	1295.3 ± 489.6	750.2	918.7	1219.5	1577.7	1920.3	131 (45.0) ^b^

Models 3 and 4 replaced milk consumed with an equal volume of FMP3+ in total children (*n* = 1122) and milk consumers (*n* = 291), respectively. Reported-T and reported-M represent the values of total children and milk consumers, respectively. Abbreviation: SD, standard deviation; NA, no Chinese EAR available. Wilcoxon signed-rank test is used to compare the intakes, ^a^ *p* < 0.01 when compare reported intakes with respective modelled intakes, i.e. reported-T vs. model 3 and reported-M vs. model 4. Paired Chi-square test is employed to compare the proportions of children below nutrient intake recommendations, ^b^ *p* < 0.05 when comparing reported with respective modelled proportions, i.e., reported-T vs. model 3 and reported-M vs. model 4.

## Supplementary Materials

The following are available online at https://www.mdpi.com/2072-6643/12/2/554/s1, Table S1: Age-specific dietary nutrient intake recommendations of 3–8 year old children according to the Chinese Dietary Reference Intakes 2013.
