# Supplementary material for: Dairy Intake Would Reduce Nutrient Gaps in Chinese Young Children Aged 3–8 Years: A Modelling Study
Source: Nutrients. 2020 Feb 20;12(2):554. doi: 10.3390/nu12020554 (PMC7071369; doi:10.3390/nu12020554)
Supplement: Supplementary file 1 [file nutrients-12-00554-s001.pdf]

Table S1. Age-specific dietary nutrient intake recommendations of 3–8 year old children according to the Chinese Dietary Reference Intakes 2013 [a].

Nutrients	EAR			AI			Lower Limit Of AMDR	PI-NCD		
	3 ≤ age < 4 years	4 ≤ age < 7 years	7 ≤ age < 8 years	3 ≤ age < 4 years	4 ≤ age < 7 years	7 ≤ age < 8 years	3 ≤ age < 8 years	3 ≤ age < 4 years	4 ≤ age < 7 years	7 ≤ age < 8 years
Carbohydrate (g/day)	120	120	120							
Carbohydrate (E%)							50			
Protein (g/day)	25	25	25							
Fat (E%) [b]				35			20			
Vitamin C (mg/day)	35	40	55							
Thiamin (mg/day)	0.5	0.6	0.8							
Riboflavin (mg/day)	0.5	0.6	0.8							
Calcium (mg/day)	500	650	800							
Phosphorus (mg/day)	250	290	400							
Magnesium (mg/day)	110	130	180							
Vitamin A (μg RAE/day)	220	260	360							
Iron (mg/day)	6	7	10							
Zinc (mg/day)	3.2	4.6	5.9							
Selenium (μg/day)	20	25	35							
Sodium (mg/day)[c]				700	900	1200		995	1200	1500
Potassium (mg/day)				900	1200	1500				

Abbreviations: EAR, estimated average requirement; AI, adequate intake; AMDR, acceptable macronutrient distribution ranges; PI-NCD, proposed intakes for preventing non-communicable chronic diseases; E, energy; RAE, retinol activity equivalents. [a] Dietary nutrient intake recommendations including EAR, AI and lower limit of AMDR were used to estimate the proportions of children with inadequate nutrient intakes, while PI-NCD of sodium was used to investigate the proportion of children with excessive sodium intake. [b] Fat (E%) inadequacy rate was assessed by compare to AI of fat (E%) for 3–4 year old children, and to lower limit of AMDR of fat (E%) for those aged 4–8 years. [c] PI-NCD of sodium for children aged 3–4 years was unavailable in the Chinese Dietary Reference Intakes 2013, which was replaced by that of 4–7 year group, adjusted by the ratio of average dietary energy intake between 3–4 and 4–7 year groups in present study.
